# *Gemella morbillorum* infective endocarditis: A case report and literature review

**DOI:** 10.1515/biol-2022-0599

**Published:** 2023-05-18

**Authors:** Xuejie Cao, Lichao Yuan

**Affiliations:** Genoxor Medical Science and Technology Inc., Shanghai, China; Department of Infectious Disease, China-Japan Friendship Hospital, No. 2, Yinghuayuan East Street, Chaoyang, Beijing 100010, China

**Keywords:** *Gemella morbillorum*, infective endocarditis, metagenome next-generation sequencing, aortic valve

## Abstract

Infective endocarditis (IE) caused by *Gemella morbillorum* is rare. Consequently, little is known about the natural course of endocarditis caused by this pathogen. This report describes the case of a 37-year-old male patient with *G. morbillorum* endocarditis. The patient was hospitalized for a fever of unknown origin. He complained of intermittent fever of unknown origin for 2 months. He had also undergone root canal therapy for pulpitis a month ago. After admission, the infectious pathogen *G. morbillorum* was identified using metagenomic next-generation sequence technology. The anaerobic blood culture bottle showed only Gram-positive cocci. Transthoracic echocardiography showed 10 mm vegetation on the aorta, which met the IE diagnostic Duke’s criteria, and the patient was diagnosed with *G. morbillorum* IE. Because no bacterial colonies were formed on the culture, the drug sensitivity test could not be conducted. Ceftriaxone anti-infective drugs are based on careful consideration of the literature and patient. Six days after antibiotic treatment in our department, the patient was discharged from the hospital in stable condition and had no adverse reactions at 1 week of follow-up. To help clinicians better understand the disease of *G. morbillorum* IE, we also reviewed and discussed the relevant cases published after 2010 when presenting the report.

## 1 Background

*Gemella morbillorum*, a Gram-positive, catalase-negative, facultatively anaerobic coccus, was given by Tunnicliff in 1917 [1]. *G. morbillorum* often behaves as commensal bacteria of the mucous membranes of the oropharynx, gastrointestinal tract, and genitourinary tract [2]. *G. morbillorum* causes a variety of infections in men, including meningitis, spondylodiscitis, pneumonia, osteomyelitis, endocarditis, and peritonitis [3,4,5,6,7]. Compared with other pathogens, *G. morbillorum* has a lower virulence. Still, delayed antimicrobial treatment of *G. morbillorum* endocarditis carries an increased risk of embolism or immune events. In severe cases, the risk of death also increases [8,9].

Infective endocarditis (IE) is a life-threatening infectious disease. The current pathogen spectrum includes *Staphylococcus aureus* and *Streptococci*, which are most of the organisms involved [10]. IE caused by *G. morbillorum* is currently rare and it is also rare in China. We present a case of a 37-year-old male diagnosed with *G. morbillorum* IE, in whom *G. morbillorum* was accurately detected by metagenomic next-generation sequence (mNGS). The patient benefited from this, received timely targeted treatment, and recovered well.

## 2 Case presentation

A 37-year-old male with a fever (38°C) presented to the fever clinics with a 2-month history of intermittent low-grade fever, chills, hunger, and headache. About a month back, he underwent root canal therapy for pulpitis without receiving antibiotic prophylaxis. He developed a fever the night after the operation and was treated with tinidazole combined with cephalosporin for empirical anti-infection. After 1 week of treatment, the temperature returned to normal but the fever recurred 2 days after stopping the medication, which was not taken seriously. At this visit to our hospital, the white blood cell (WBC) counts were 9.67 × 10^9^/L, neutrophil counts were 7.74 × 10^9^/L, and the C-reactive protein (CRP) level was 51.01 mg/L. The value of the N-terminal B-type natriuretic peptide precursor (NT-proBNP) was as high as 159 pg/mL, so the patient was transferred to our department for further diagnosis and treatment.

He reported eating normally, regular bowel movements. He did not suffer from hypertension, diabetes, heart disease, cerebrovascular disease, hepatitis, history of tuberculosis, history of trauma, blood transfusion, intrathoracic, intracardiac procedures, or gastrointestinal tract pathology. He also did not have any history of intravenous drug use or smoking.

### 2.1 Investigations

On physical examination, the patient was afebrile and sane. His blood pressure was 150/45 mmHg, with a heart rate of 104 bpm. There was no bump in the precordial area, the cardiac boundary was not enlarged, the heart sound was strong, the rhythm was uniform, and the level 2 wind-blowing diastolic murmur could be understood in the precordial area without a pericardial murmur. His skin was normal without rashes or subcutaneous bleeding, and his ophthalmoscopic examination was negative. Hearing and smell were normal. Oral hygiene was good, and the oropharynx was very healthy. Auscultation of the chest revealed clear lung fields bilaterally without any adventitious sounds. Physical examination revealed no abnormality in the chest, lungs, abdomen, liver, spleen, limbs, or intestines. It may be due to the brief duration of the disease that there were no common endocarditis signs, such as Osler node, Janeway lesion, or Roth spot.

**Figure 1:**
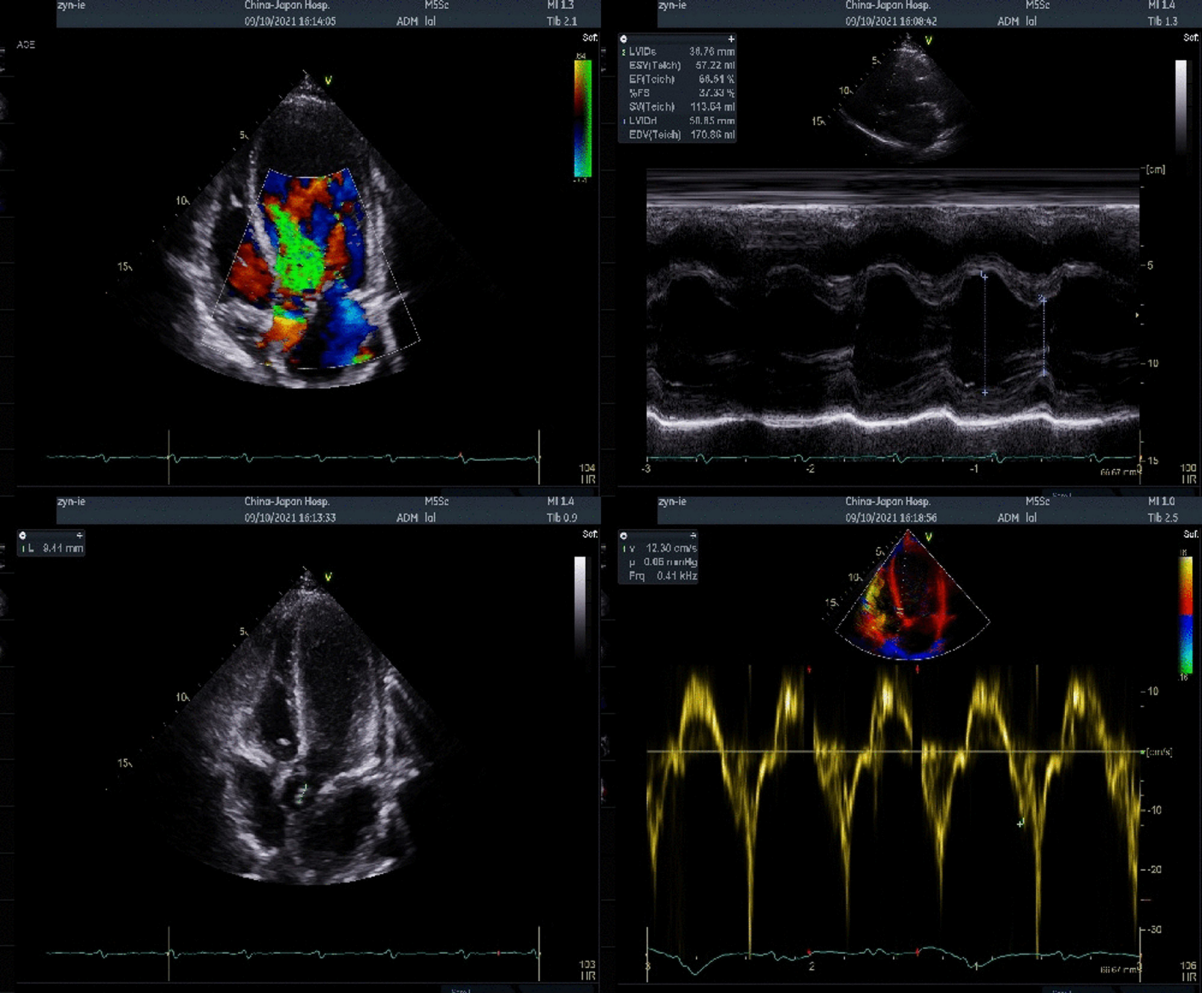
Transthoracic echocardiography (including tissue Doppler) showing aortic valve bivalve malformation, abnormal echo, and severe regurgitation left atrial dilatation, and moderate mitral regurgitation, with LVEF of 66%.

Laboratory workup was notable for a hemoglobin level of 123 g/L, hypersensitive C-reactive protein (HS-CRP) of 57.65 mg/L, and an erythrocyte sedimentation rate of 58 mm/h on the day of admission to our department. We followed the blood routine examinations and cardiac function indexes on the second and third days. The WBC counts were 9.65 × 10^9^/L and 9.91 × 10^9^/L, neutrophil counts were 7.81 × 10^9^/L and 7.42 × 10^9^/L, and CRP levels were 71.65 and 48 mg/L. The NT-proBNP levels reached as high as 2,173 pg/mL. Transthoracic echo was notable for the aortic valve bivalve malformation, abnormal echo, and severe regurgitation left atrial dilatation, moderate mitral regurgitation, and left ventricular ejection fraction (LVEF) of 66% (Figure 1). So, aortic valve vegetation was considered. The longest was about 10 cm, and IE was highly suspected. Chest computed tomography (CT) examination showed no obvious abnormality.

On pathogen examination, we timely collected blood samples from patients for culture. To obtain etiologic results quickly, we performed mNGS detection with the patient’s consent. On the second day, the results of feedback on specific pathogen tests showed that the 1,3-β-D-glucan (G) and galactomannan (GM) tests were negative. The respiratory-tract inspection was negative, and the Coxsackie virus antibody was negative. EBV and CMV were identified by multiplex polymerase chain reaction (PCR), and the results were also negative. The serological tests were negative, and the rubella virus and *Toxoplasma gondii* serological tests were also negative. The mNGS results from feedback within 24 h showed that 63 reads of *G. morbillorum* were detected in the cfDNA of blood samples (Table 1). On the fourth day, the anaerobic blood culture bottle showed Gram-positive cocci, so the possibility of *G. morbillorum* was high. At this point, our patient met “diagnosed” endocarditis per Duke’s criteria, and the pathogen was confirmed as *G. morbillorum*.

**Table 1:** Results of mNGS

Types	Species		Genus	
	Name	Reads	Name	Reads
Gram positive bacteria	*Gemella morbillorum*	63	*Gemella*	69


**Informed consent**: Informed consent has been obtained from all individuals included in this study.
**Ethical approval:** The research related to human use has complied with all the relevant national regulations, institutional policies and is in accordance with the tenets of the Helsinki Declaration, and has been approved by the authors’ institutional review board or equivalent committee.

### 2.2 Methods of mNGS

According to the Illumina next-generation sequencing assay manual, blood samples were processed in a medical laboratory (Nextseq CN500). The general detection process was as follows:

1. A total of 200 μL of plasma was used to extract the nucleic acid for cfDNA libraries.
2. Nextseq CN500 was used to perform sequencing after the library was validated by the 2100 Bioanalyzer system (Agilent Technologies, Inc.) and RT-PCR.
3. Sequence reads were classified according to their origin by a developed bioinformatics pipeline.

The bioinformatics process mainly included the following steps:

1. Remove low-quality reads.
2. Host reads were subtracted.
3. The remaining reads were aligned to the reference database, composed of multiple public sequence resources of bacteria, viruses, and fungi.

### 2.3 Differential diagnosis

The patient showed fever, so we checked whether it was rheumatic fever, sepsis, systemic lupus erythematosus, and autoimmune diseases. The patient was excluded for rheumatic fever because there was no history of upper respiratory tract infection, joint pain, or rheumatic heart disease. Similarly, this patient did not meet the clinical symptoms of sepsis, i.e., high fever, chills, and obvious respiratory, digestive, urinary, and other infectious symptoms. In patients with systemic lupus erythematosus, pericarditis is the most common cardiac involvement; valve involvement is less, and most patients are female and excluded. Regarding autoimmune diseases, the anti-nuclear antibody spectrum of the patients was examined and all of them were negative. Then, we improved the results of echocardiography and etiological examination as soon as possible to ascertain whether it was IE.

### 2.4 Treatment

Once a transthoracic echo revealed suspected endocarditis, our patient was empirically treated with ceftriaxone (2 g every 24 h was initiated) and netilmicin (2 g every 24 h was initiated). This decision was supported when the mNGS resulted in *G. morbillorum*, and blood cultures grew Gram-positive cocci. Because the drug sensitivity information could not be obtained through culture, we continued to use ceftriaxone for anti-infection treatment in combination with the literature and the patient’s situation. Simultaneously with the anti-infection, as the patient showed rapid heart rate, enlarged left atrium, and elevated NT-proBNP, we considered that the IE might involve the valve to cause heart failure, and torasemide diuresis was given to correct heart failure. Due to the increased activity of aortic vegetation in patients, we should be vigilant against systemic embolism caused by the shedding of vegetation.

### 2.5 Outcome

After 6 days of treatment in our department, the patient’s condition appeared stable without any particular discomfort. The general situation worked well. The vital signs showed that the body temperature was 36.4°C, the blood pressure was 140/60 mmHg, the heart rate was 84 bpm, and the blood routine test results showed that the fluctuation range of various indicators was small. The CRP decreased to 21.83 mg/L. After only 1 week in the hospital, the patient firmly asked to be discharged. Blood was collected and cultured the day before discharge again, and sterile growth occurred 5 days later. No antibiotics were prescribed at discharge. We repeatedly explained the necessity of continued hospitalization and suggested that the patient receive cardiac surgical treatment as soon as possible. On the second day after discharge, the patient was sent to a top-grade hospital in Beijing for surgical treatment. At the follow-up, the patient was receiving preoperative intravenous antibiotic therapy. Unfortunately, we could not obtain the antibiotic information.

## 3 Discussion

*G. morbillorum* is one of the rare causative microorganisms of endocarditis. We herein report a case of IE in a patient with an aortic valve caused by *G. morbillorum*. Based on modified Duke’s criteria [11], the patient was diagnosed with IE. In addition to the case description, we reviewed 16 cases of *G. morbillorum* endocarditis that were published between 2010 and 2021 (Table 2) [8,9,12,13,14,15,16,17,18,19,20,21,22,23,24,25]. Integrating patient information and discussion were required to expect a more profound and systematic understanding of this rare case.

**Table 2:** Reported cases of IE caused by *Gemella morbillorum* from 2010 to 2021

References	Country	Age/gender	Pathogen detection	The duration of antimicrobial therapy	Valve replacement	Valve type	Valves involved	Risk factors	Survival outcome
Desai et al., 2021 [12]	USA	72/male	MALDI-TOF	After 2 weeks of vancomycin treatment, it was changed to cefazolin treatment for 4 weeks	Mitral and aortic valve	Natural valve	Mitral, aorticn and pulmonary valves	Not explained	Alive
Tanveer et al., 2021 [13]	USA	48/male	Blood culture	Ceftriaxone was initiated for a 6-week duration	Mitral valve	Natural valve	Mitral valve	This patient did not have known predisposing conditions	Alive
Patel et al., 2021 [14]	USA	56/female	Blood culture	Ceftriaxone was initiated for a 6-week duration	No	Prosthetic valve	Aortic valve	Poor dentition and underlying cardiac pathologies	Alive
Dogan et al., 2020 [15]	Turkey	37/male	MALDI-TOF	Ceftriaxone was initiated for a 6-week duration	Mitral and aortic valve	Natural valve	Aortic valve	Bicuspid aorta	Alive
Spaeth et al., 2020 [16]	USA	23/male	MALDI-TOF	Gentamicin and ampicillin for 31 days	Mitral and aortic valve	Natural valve	Aortic and mitral valves	Dental caries	Alive
Lee et al., 2019 [17]	USA	33/male	Not explained	Not explained	Pulmonary and aortic valve	Natural valve	Aortic and pulmonary valves	Congenital heart defect	Not explained
Rehman et al., 2019 [18]	USA	34/male	Blood culture	Not explained	Pulmonary and aortic valve	Natural valve	Aortic and pulmonary valves	This patient did not have known predisposing conditions	Not explained
Li et al., 2017 [19]	China	28/male	Blood culture	Ceftriaxone for 5 weeks and vancomycin for 1 week	Pulmonary valve	Natural valve	Pulmonary valve	Congenital ventricular septal defect, atrial septal defect, and double-chambered right ventricle	Alive
Shinha, 2017 [20]	USA	37/male	Blood culture	Not explained	Not explained	Natural valve	Aortic valve	A history of intravenous drug abuse	Not explained
Rosa et al., 2015[8]	Brazil	72/male	Blood culture	After 1 day of ceftriaxone with gentamicin, it was changed to penicillin G with gentamicin treatment for 27 days	Mitral valve	Natural valve	Mitral valve	Previous coronary artery bypass graft surgery	Deceased
Constantinos et al., 2015 [21]	Cyprus	80/female	Blood culture	Ceftriaxone for 6 weeks and gentamycin for 2 weeks	No	Prosthetic valve	Tricuspid valve	History of aortic valve replacement	Alive
Ural et al., 2014 [22]	Turkey	67/male	Blood culture	After 3 weeks of ampicillin/sulbactam with gentamicin, it was changed to meropenem and vancomycin treatment for 1 week	Not explained	Natural valve	Not explained	Not explained	Alive
Shahani, 2014 [23]	USA	73/male	16S rRNA	Penicillin G with gentamicin for 6 weeks	Aortic valve	Prosthetic valve	Aortic valve	Poor dentition and underlying cardiac pathologies	Alive
Kolhari et al., 2014 [24]	India	34/female	Blood culture	Crystalline penicillin and levofloxacin for 2 weeks	Mitral valve	Natural valve	Mitral valve	Asymptomatic obstructive hypertrophic cardiomyopathy and had undergone dental extraction	Alive
Taimur et al., 2010 [25]	Pakistan	31/female	Blood culture	Ceftriaxone for 6 weeks and gentamycin for 2 weeks	No	Natural valve	Aortic valve	A congenitally bicuspid aortic valve and had undergone repair of a large aortic aneurysm	Alive
Massoure et al., 2010 [9]	Djibouti	22/male	Blood culture	Amoxicillin and gentamycin (no treatment time)	No	Natural valve	Aortic valve	Dental caries and periodontitis	Deceased
This case	China	37/male	mNGS and blood culture	Netilmycin with cefatriaxone for 1 week	Not explained	Natural valve	Aortic valve	Dental surgery	Alive

Retrospective cases revealed that aortic valves were affected in many cases, followed by the mitral valve. In contrast, the tricuspid valve and pulmonary valve were rarely involved. Tricuspid IE caused by *G. morbillorum* is extremely rare, and we only retrieved one case. Compared with a prosthetic valve, natural valves are more vulnerable.

Current trends in *G. morbillorum* IE from retrieved case reports showed that congenital heart disease is the most common risk factor (5/16). Apart from this, underlying conditions such as poor dentition and dental surgery (5/16), cardiac valvular disease (2/16), history of intravenous medication (1/16), and history of cardiac surgery were associated with human infection by *G. morbillorum*. In our case, the patient had an intermittent fever before 2 months and had dental surgery a month back. Therefore, dental surgery may be one of the factors causing this infection. *Gemella* spp. can adhere to the oral cavity surfaces using adherent proteins [26]. Therefore, the oral cavity is an ideal medium for *Gemella*. Furthermore, *Gemella* shares the structural features of IgA proteases, which aid in human IgA destruction [27]. It also explains why dental surgery or oral health is a common risk factor for *G. morbillorum* IE. Our case report highlights the importance of recognizing oral health in diagnosing and treating endocarditis.

In addition, according to our review of case reports, 2/16 patients had a final clinical outcome of death. This fact underscores the potential virulence of *G. morbillorum* in the setting of IE. The ability of this bacterium to destroy the cardiac valve tissue contributes to the rapid deterioration of patients with endocarditis, culminating in cardiogenic shock and even death. For this reason, we recommend maintaining a high *G. morbillorum* infection index in difficult-to-identify cases to facilitate early detection and accurate identification at the etiological diagnosis level.

In our summary cases, in 11/16 patients, the pathogen of *G. morbillorum* was identified through a blood culture. The gold standard for the etiological detection of IE is blood culture. However, *G. morbillorum* is not always considered first by clinicians, limiting the use of culture in diagnosis. Unlike culture, mNGS, with a high sensitivity and good timeliness and specificity, can unbiasedly detect almost all organisms in the sample without an *a priori* hypothesis and has been extensively used for the clinical diagnosis of various infections with satisfactory performance. This case is also the first known patient where, with an mNGS-assisted diagnosis of *G. morbillorum*, IE was clinically confirmed. It shows rapid and accurate characteristics in etiology diagnosis. Our finding indicated that mNGS could be an alternative for early diagnosis of *G. morbillorum*.

The treatment of *G. morbillorum* is either medical therapy or surgical replacement of the valve. Most patients recovered with combined treatment of antibiotics and surgery. Most *G. morbillorum* IE cases were cured with ceftriaxone, with gentamicin and vancomycin added empirically in a few reports. Because the blood culture result of our case showed only Gram-positive cocci, no drug sensitivity test was conducted. The antibiotic protocol formulation resulted from carefully considering the literature and the patient’s situation.

## 4 Conclusions

Clinicians need to be conscious that *G. morbillorum* can occasionally cause IE. The organisms must be considered, especially if the patient has underlying or predisposing conditions. Metagenome sequencing technology is a good choice in assisting the rapid clinical identification of IE pathogens.
